# *MAPT* haplotype–stratified GWAS reveals differential association for AD risk variants

**DOI:** 10.1002/alz.12099

**Published:** 2020-05-13

**Authors:** Samantha L. Strickland, Joseph S. Reddy, Mariet Allen, Aurelie N'songo, Jeremy D. Burgess, Morgane M. Corda, Travis Ballard, Xue Wang, Minerva M. Carrasquillo, Joanna M. Biernacka, Gregory D. Jenkins, Shubhabrata Mukherjee, Kevin Boehme, Paul Crane, John S. Kauwe, Nilüfer Ertekin‐Taner, Alzheimer's Disease Genetics Consortium

**Affiliations:** ^1^ Department of Neuroscience Mayo Clinic Jacksonville Florida USA; ^2^ Department of Health Sciences Research Mayo Clinic Jacksonville Florida USA; ^3^ Department of Health Sciences Research Mayo Clinic Rochester Minnesota USA; ^4^ University of Washington Seattle Washington USA; ^5^ Brigham Young University Provo Utah USA; ^6^ Department of Neurology Mayo Clinic Jacksonville Florida USA

**Keywords:** Alzheimer's disease, case‐control studies, co‐expression networks, differential gene expression, haplotype‐stratified genome‐wide association, *MAPT*

## Abstract

**Introduction:**

*MAPT* H1 haplotype is implicated as a risk factor for neurodegenerative diseases including Alzheimer's disease (AD).

**Methods:**

Using Alzheimer's Disease Genetics Consortium (ADGC) genome‐wide association study (GWAS) data (*n* = 18,841), we conducted a *MAPT* H1/H2 haplotype–stratified association to discover *MAPT* haplotype–specific AD risk loci.

**Results:**

We identified 11 loci—5 in H2‐non‐carriers and 6 in H2‐carriers—although none of the *MAPT* haplotype–specific associations achieved genome‐wide significance. The most significant H2 non‐carrier–specific association was with a *NECTIN2* intronic (*P* = 1.33E‐07) variant, and that for H2 carriers was near *NKX6‐1* (*P* = 1.99E‐06). The *GABRG2* locus had the strongest epistasis with *MAPT* H1/H2 variant rs8070723 (*P* = 3.91E‐06). Eight of the 12 genes at these loci had transcriptome‐wide significant differential expression in AD versus control temporal cortex (*q* < 0.05). Six genes were members of the brain transcriptional co‐expression network implicated in “synaptic transmission” (*P* = 9.85E‐59), which is also enriched for neuronal genes (*P* = 1.0E‐164), including *MAPT*.

**Discussion:**

This stratified GWAS identified loci that may confer AD risk in a *MAPT* haplotype–specific manner. This approach may preferentially enrich for neuronal genes implicated in synaptic transmission.

## 1 INTRODUCTION

Tauopathies, a class of neurodegenerative disorders, are characterized by neurofibrillary tangles (NFTs) in the brain due to pathological aggregation of hyperphosphorylated microtubule‐associated protein tau (MAPT), encoded by the *MAPT* gene on chromosome 17q21.3. Tau tangles are present in the brains of patients with progressive supranuclear palsy (PSP), corticobasal degeneration (CBD), Pick disease, dementia pugilistica, frontotemporal dementia, and parkinsonism linked to chromosome‐17 (FTDP‐17 or frontotemporal lobar degeneration with tau pathology (FTLD‐tau)), and other neurodegenerative diseases, including Alzheimer's disease (AD), the most prevalent tauopathy and cause of dementia.^1^ In addition to senile plaques composed primarily of extracellular amyloid beta (Aβ), the presence of NFTs is a hallmark of AD pathology.

*MAPT* variants have been implicated in the etiology and pathogenesis of multiple neurodegenerative diseases. The discovery of multiple *MAPT* mutations in FTDP‐17 provided some of the first evidence that changes in tau alone could cause neurodegenerative disease. The FTDP‐17 splice‐site mutations within *MAPT* demonstrated that an imbalance in the ratio of 3R and 4R tau isoforms is sufficient to cause disease.^2^, ^3^, ^4^ Further association studies revealed that the locus can be divided into two major haplotypes: H1 and H2. *MAPT* falls within the largest known block of linkage disequilibrium (LD) in the human genome, spanning ≈1.8 Mb. There is a 900 kb inversion of the H2 haplotype with respect to the H1 haplotype, covering a region encompassing several genes, including MAPT, IMP5, CRHR1, and NSF. The inversion results in a reduced recombination between the inverted H2 and non‐inverted H1 haplotypes.

The common *MAPT* haplotype H1 shows robust association with risk for the primary tauopathies PSP^5^ and CBD,^6^ as well as Parkinson disease (PD), which is not considered as a tauopathy.^7^ *MAPT* H1 haplotype–tagging single‐nucleotide polymorphisms (SNPs) were identified among the top PSP^8^ and PD genome‐wide association study (GWAS)^9^ signals. In addition, *MAPT* H1 haplotype shows considerable variation^10^, ^11^ and leads to H1‐ subhaplotypes, where H1c, has been implicated in the risk of PSP, CBD, AD, and PD.^12^, ^13^ *MAPT* H2 haplotype has been associated with reduced risk for several neurodegenerative disorders.^14^, ^15^

Although *MAPT* is a compelling candidate for neurodegenerative disease susceptibility, evidence of association of AD with the *MAPT* H1 and H2 haplotypes have produced equivocal results.^12^, ^16^, ^17^ This may in part be due to limited sample sizes, and therefore limited power for most *MAPT* haplotype association studies in AD. In a large study from Genetic and Environmental Risk for Alzheimer's Disease (GERAD1) consortium,^18^ the *MAPT* H2 haplotype–tagging variant was found to have association with reduced AD risk. In a study of >20,000 individuals from Mayo Clinic and the Alzheimer's Disease Genetics Consortium (ADGC), we identified associations with both reduced AD risk and reduced brain *MAPT* levels with the H2 haplotype.^14^ In addition, a recent meta‐analysis pooling 39 studies in AD again demonstrated association of reduced AD risk with the *MAPT* H2 haplotype.^15^

**HIGHLIGHTS**

- Microtubule‐associated protein tau gene (*MAPT*) H1 and H2 carriers have discordant Alzheimer's disease (AD) risk loci, most of which are novel.
- Many of the genes at these loci are differentially expressed in AD brains.
- The *MAPT* haplotype–stratified approach identified genes in synaptic transmission networks.

**RESEARCH IN CONTEXT**

1. Systemic review: Comprehensive review of the literature shows that the microtubule‐associated protein tau gene (*MAPT*) is a strong candidate for neurodegenerative disease susceptibility. The *MAPT* H2 haplotype is associated with lower Alzheimer's disease (AD) risk in large cohorts and lower brain *MAPT* levels.
2. Interpretation: We hypothesized that AD risk variants exhibit *MAPT* haplotype–dependent association. Through haplotype‐stratified association analyses using data from the Alzheimer's Disease Genetics Consortium (ADGC) on 18,841 participants, we identified 11 loci with *MAPT* H1– or H2–specific AD risk association. Eight genes at these loci had significant differential expression in AD versus control brains. Six genes were members of the neuronal‐enriched brain transcriptional co‐expression network implicated in synaptic transmission.
3. Future directions: Replication of *MAPT* haplotype–stratified associations should be sought in larger cohorts. Candidate genes from this study should be evaluated for the presence of functional variants that may influence tau‐related outcomes. Emerging larger cohorts with multiomics data and generation of more complex model systems will enable these studies.

In the current study, we sought to further elucidate the role of *MAPT* H1 and H2 haplotypes in AD susceptibility by leveraging the genome‐wide genotype data available from the sizable ADGC case‐control series. Using haplotype‐stratified analyses, we tested the hypothesis that AD risk variants exhibit *MAPT* haplotype–dependent association and may therefore potentially identify novel AD risk variants with implications for functional pathways. Analysis of a stratum with a more homogeneous AD risk profile with respect to *MAPT* H1/H2 haplotype may help uncover loci that have differential influence on AD risk in a MAPT context‐specific manner. For example, given the association of *MAPT* H2 with lower brain *MAPT* levels, it is plausible that those loci with *MAPT* H2–specific associations harbor genes that influence neurodegeneration via pathways that are not dependent on elevated tau. In contrast, AD risk associations in H2 non‐carriers may enrich for loci that confer risk in a tau level‐dependent fashion.

Our approach herein is akin to pursuing GWAS in an apolipoprotein E gene (*APOE*)–stratified fashion.^19^ Although *MAPT* haplotypes tested to date in the literature clearly have smaller effect sizes than that of *APOE* genotypes for AD risk, it is nonetheless worthwhile to pursue this *MAPT* haplotype–stratified analysis not only because of its potential to identify novel loci but also because of the plethora of data implicating tau in AD in functional studies.^20^ In this study, we evaluated known International Genomics of Alzheimer's Project (IGAP) AD^21^ risk loci in a *MAPT* haplotype–stratified analysis, which did not reveal evidence of *MAPT* haplotype–specific associations. We also identified novel AD risk loci with association only in *MAPT* H2 carriers (six loci) or H2‐non‐carriers (five loci). We characterize genes near both the known and the new loci for their expression levels and co‐expression networks in a brain transcriptome data set of AD and control temporal cortex.^22^, ^23^ Our findings, which require replication in larger cohorts, suggest that *MAPT* haplotype–stratified GWAS may identify novel loci, and that genes at these loci are expressed predominantly within neuron‐enriched networks implicated in synaptic transmission.

## 2 METHODS

### 2.1 Study populations

The ADGC data were used for this study. Subjects available through the ADGC have been described previously and are available through ftps from the UPENN server (alois.med.upenn.edu).^24^, ^25^, ^26^, ^27^ The data set included all the covariates required for the analysis and all actual and imputed genotypes. Post–quality control (post‐QC) data for both the actual and imputed genotypes and designations for all the sub‐cohorts included in the ADGC data were obtained. The demographics detailing each cohort and stratified group are described in Table S1. The cohort for the expression analysis was the Mayo Clinic RNAseq data set.^22^ Detailed methods are provided in Supplementary Methods.

### 2.2 AD risk association analysis

Variants were evaluated for association with AD using multivariable logistic regression implemented in PLINK.^28^ Both joint (full data set of 21 cohorts analyzed jointly, adjusting for cohort) and meta‐(separate cohorts) analyses were performed. For the meta‐analysis, a random effects method was adopted due to presence of heterogeneity, *I* ^2^ > 25.^29^ An additive model for the minor alleles determined in the unstratified data set was applied with the covariates age, sex, and PC1‐3 (principal components 1‐3) used throughout all models. A second model using the additional *APOE* covariate in the joint and meta‐analyses was also evaluated. Two IGAP loci variants rs4147929 and rs9331896 were filtered out of the original data set due to the QC procedures described previously.^27^ They were evaluated separately for the joint analyses using the same method above. Meta analyses could not be performed for rs4147929 and rs9331896 due to their absence from the original data set. To generate forest plots for the variants of interest, meta‐analysis was performed in R^30^ with the Metafor package^31^ using the random effects method with DerSimonian Laird estimator for the variance between studies/cohorts. To determine the joint effect of the tested SNPs and *MAPT* haplotypes on AD risk, we also performed a bivariate analysis, described in Supplementary Methods.

### 2.3 Epistasis analysis

SNP–SNP interactions of epistasis between each of the 3,067,502 SNPs and the H2 tagging variant rs8070723‐G were conducted. Two models were evaluated for the H2 tagging variant, a carrier model (H1H1 and H1H2+H2H2) and a dosage model (H1H1, H1H2, and H2H2). The analysis was performed by creating a distance matrix in PLINK between each SNP and rs8070723‐G. Two general linear models (with SNPx rs8070723‐G interaction and without interaction) were executed using age, sex, ADGC cohort, and PC1‐3 as covariates followed by an analysis of variance (ANOVA) to assess the significance between the models using the chi‐square method as implemented in R.

### 2.4 Gene expression analyses

Differential gene expression and co‐expression network analyses were conducted as previously published.^23^, ^32^ For each gene, multiple linear regression was performed in which normalized gene expression was the dependent variable, diagnosis (AD vs control) was the independent variable of primary interest and sex, flowcell, age at death, RNA integrity number (RIN), and center from which the samples were obtained were the covariates. Weighted Gene Co‐Expression Network Analysis (WGCNA) was utilized to identify brain co‐expression networks and test their associations with AD as we reported previously.^23^

### 2.5 Visuals

The figures were generated using the lattice^33^ and metafor packages in R and Inkscape (www.inkscape.org).

## 3 RESULTS

### 3.1 MAPT haplotype–specific association analysis at known AD risk loci

Using genome‐wide genotype data from 21 cohorts within ADGC, we tested the hypothesis that AD risk variants exhibit *MAPT* haplotype–specific association. Following QC measures, approximately 3 million variants with a minor allele frequency (MAF) ≥0.02, and all index variants identified by the IGAP consortium^21^ were retained for analysis and evaluated for *MAPT* haplotype–specific association. *MAPT* H2 haplotype tagging allele rs8070723‐G was used to stratify study participants into H2 carriers (H1H2+H2H2: 3631 cases, 3729 controls) and H2 non‐carriers (H1H1: 5958 cases, 5523 controls). The demographics of the cohorts of the H2 carriers and non‐carriers are described in Table S1.

GWAS analyses with AD were performed using joint and meta‐analyses. There was no evidence of population stratification based on the quantile‐quantile plots (QQ plots) (Figure S1) and the genomic inflation factors of 1.04, 1.04, and 1.01 for the unstratified, H2 non‐carrier, and H2 carrier joint analyses, respectively. The joint and meta‐analyses yielded similar results with respect to genomic inflation. Likewise, the addition of *APOE* as a covariate did not significantly alter the results. We adopted the joint analysis approach without *APOE* covariate as the primary model.

We first evaluated the previously reported IGAP^21^ AD risk variants to determine if they exhibit *MAPT* haplotype–specific association. As expected, the unstratified analysis results were similar to those reported in the IGAP study, albeit with reduced significance due to the smaller cohort size (Figure S2, Table 1). IGAP index variants had similar direction of AD risk in both the H2 non‐carrier and H2 carrier analyses. To determine whether any of these variants had a significantly different effect on AD risk based on the *MAPT* haplotype, we performed epistasis analysis with the *MAPT* H1/H2 haplotype tagging variant. Only two IGAP variants, rs10948363 (*CD2AP*) and rs1476679 (*ZCWPW1/PILRB*), showed a trend of epistasis (uncorrected *P* < 0.05) with the *MAPT* H1/H2 haplotype–tagging variant (Table 1); however, the odds ratios (ORs) for both variants were in the same direction with overlapping 95% confidence intervals (CIs). In summary, we found no strong evidence of *MAPT* haplotype–specific association for the reported IGAP AD risk SNPs.

**TABLE 1:** IGAP loci associations stratified by H2 non‐carriers and H2 carriers

						Unstratified^f^						H2 non‐carriers^f^						H2 carriers^f^						
SNP^a^	CHR^c^	Position^c^	Closest gene^d^	Major/Minor^c^	MAF^e^	N_j_ ^g^	OR_J_ (95% CI)^g^	*P*_J_ ^g^	N_M_ ^h^	OR_m_ ^h^	P_M_ ^h^	N_j_ ^g^	OR_J_ (95% CI)^g^	P_J_ ^g^	N_M_ ^h^	OR_m_ ^h^	P_M_ ^h^	N_j_ ^g^	OR_J_ (95% CI)^g^	P_J_ ^g^	N_M_ ^h^	OR_m_ ^h^	P_M_ ^h^	Epistatis P^i^
rs6656401	1	207692049	CR1	G/A	0.183	17833	1.15 (1.08‐1.21)	2.37E‐06	21	1.15	1.37E‐06	10854	1.13 (1.05‐1.21)	9.82E‐04	21	1.14	7.48E‐04	6979	1.17 (1.07‐1.28)	7.45E‐04	21	1.17	1.36E‐03	5.37E‐01
rs6733839	2	127892810	BIN1;CYP27C1	C/T	0.394	14091	1.23 (1.17‐1.3)	4.39E‐16	21	1.23	1.89E‐10	8627	1.23 (1.15‐1.31)	2.64E‐10	21	1.23	1.25E‐09	5464	1.24 (1.14‐1.35)	3.43E‐07	21	1.27	6.23E‐08	8.38E‐01
rs35349669	2	234068476	INPP5D	C/T	0.482	17273	1.04(1‐1.09)	7.35E‐02	21	1.04	7.39E‐02	10535	1.04 (0.99‐1.11)	1.37E‐01	21	1.05	9.74E‐02	6738	1.03 (0.96‐1.11)	3.69E‐01	21	1.02	5.38E‐01	8.82E‐01
rs190982	5	88223420	MEF2C‐AS1	A/G	0.384	16317	0.94 (0.9‐0.98)	9.25E‐03	21	0.93	5.87E‐03	9911	0.93 (0.88‐0.99)	2.53E‐02	21	0.92	1.03E‐02	6406	0.95 (0.88‐1.02)	1.78E‐01	21	0.96	2.87E‐01	7.69E‐01
rs9271192	6	32578530	HLA‐DRB1;HLA‐DQA1	A/C	0.272	18208	1.1 (1.05‐1.15)	1.51E‐04	21	1.10	2.37E‐03	11080	1.14 (1.07‐1.21)	7.56E‐05	21	1.14	1.85E‐03	7128	1.04 (0.96‐1.13)	2.92E‐01	21	1.05	2.73E‐01	1.02E‐01
rs10948363	6	47487762	CD2AP	A/G	0.274	18827	1.09 (1.04‐1.14)	5.18E‐04	21	1.09	5.22E‐04	11476	1.05 (0.98‐1.11)	1.56E‐01	21	1.05	1.47E‐01	7351	1.16 (1.07‐1.25)	1.60E‐04	21	1.16	6.81E‐04	3.47E‐02
rs2718058	7	37841534	GPR141;NME8	A/G	0.364	18336	0.93 (0.89‐0.98)	2.81E‐03	21	0.94	7.69E‐02	11182	0.94 (0.89‐1)	4.77E‐02	21	0.97	3.96E‐01	7154	0.92 (0.86‐0.99)	2.78E‐02	21	0.92	2.50E‐02	6.38E‐01
rs1476679	7	100004446	ZCWPW1/PILRB	T/C	0.280	18617	0.92 (0.88‐0.97)	1.22E‐03	21	0.92	1.73E‐03	11346	0.88 (0.83‐0.94)	7.06E‐05	21	0.88	8.06E‐05	7271	0.99 (0.92‐1.07)	7.82E‐01	21	0.98	7.21E‐01	2.20E‐02
rs11771145	7	143110762	EPHA1‐AS1	G/A	0.334	17291	0.93 (0.89‐0.97)	1.97E‐03	21	0.93	4.22E‐03	10525	0.93 (0.88‐0.99)	1.85E‐02	21	0.93	2.87E‐02	6766	0.93 (0.86‐1)	4.27E‐02	21	0.93	6.38E‐02	8.76E‐01
rs28834970	8	27195121	PTK2B	T/C	0.363	18538	1.13 (1.08‐1.18)	1.68E‐07	21	1.13	1.64E‐07	11305	1.14 (1.07‐1.2)	1.48E‐05	21	1.14	1.67E‐04	7233	1.12 (1.04‐1.2)	3.15E‐03	21	1.12	3.42E‐03	6.97E‐01
rs9331896^b^	8	27467686	CLU	T/C	0.377	16160	0.90 (0.86‐0.95)	2.45E‐05	NA	NA	NA	9842	0.91 (0.85‐0.96)	1.36E‐03	NA	NA	NA	6318	0.90 (0.84‐0.98)	9.41E‐03	NA	NA	NA	9.87E‐01
rs10838725	11	47557871	CELF1	T/C	0.316	18596	1.04 (0.99‐1.09)	1.25E‐01	21	1.04	9.93E‐02	11334	1.04 (0.98‐1.11)	1.63E‐01	21	1.04	2.03E‐01	7262	1.03 (0.96‐1.11)	4.20E‐01	21	1.04	2.99E‐01	7.52E‐01
rs983392	11	59923508	MS4A2;MS4A6A	A/G	0.398	18095	0.87 (0.84‐0.91)	2.44E‐09	21	0.87	5.14E‐09	11017	0.87 (0.83‐0.93)	3.61E‐06	21	0.87	3.55E‐06	7078	0.87 (0.81‐0.93)	1.11E‐04	21	0.86	4.95E‐03	9.28E‐01
rs10792832	11	85867875	PICALM;EED	G/A	0.355	18651	0.88 (0.84‐0.92)	7.90E‐09	21	0.88	9.90E‐09	11368	0.89 (0.84‐0.94)	3.32E‐05	21	0.89	7.71E‐05	7283	0.86 (0.8‐0.93)	5.00E‐05	21	0.85	4.90E‐05	5.56E‐01
rs11218343	11	121435587	SORL1	T/C	0.039	18816	0.76 (0.68‐0.84)	7.44E‐07	21	0.75	9.94E‐07	11465	0.8 (0.69‐0.92)	1.46E‐03	21	0.80	2.20E‐03	7351	0.69 (0.58‐0.83)	5.43E‐05	18	0.69	1.34E‐04	2.26E‐01
rs17125944	14	53400629	FERMT2	T/C	0.093	18809	1.12 (1.04‐1.21)	2.75E‐03	21	1.13	2.59E‐02	11459	1.11 (1.01‐1.22)	3.68E‐02	21	1.09	1.84E‐01	7350	1.15 (1.02‐1.29)	2.38E‐02	21	1.15	2.32E‐02	5.89E‐01
rs10498633	14	92926952	SLC24A4	G/T	0.217	18720	0.92 (0.88‐0.97)	2.27E‐03	21	0.93	5.80E‐03	11409	0.9 (0.84‐0.96)	1.45E‐03	21	0.90	2.50E‐03	7311	0.96 (0.88‐1.04)	3.27E‐01	21	0.97	5.18E‐01	2.21E‐01
rs8093731	18	29088958	DSG2	C/T	0.010	18683	0.94 (0.76‐1.16)	5.49E‐01	20	1.01	9.39E‐01	11389	0.9 (0.69‐1.18)	4.62E‐01	18	0.84	2.92E‐01	7294	0.99 (0.72‐1.38)	9.70E‐01	15	1.14	6.31E‐01	6.37E‐01
rs4147929^b^	19	1063443	ABCA7	G/A	0.171	16429	1.14 (1.07‐1.21)	4.60E‐05	NA	NA	NA	10002	1.09 (1.01‐1.18)	2.97E‐02	NA	NA	NA	6427	1.21 (1.09‐1.33)	1.61E‐04	NA	NA	NA	1.34E‐01
rs3865444	19	51727962	CD33	C/A	0.303	18707	0.91 (0.87‐0.96)	1.78E‐04	21	0.91	1.70E‐04	11395	0.92 (0.87‐0.98)	8.80E‐03	21	0.92	1.14E‐02	7312	0.9 (0.84‐0.97)	6.84E‐03	21	0.89	3.35E‐03	6.27E‐01
rs7274581	20	55018260	CASS4	T/C	0.084	18599	0.87 (0.8‐0.94)	3.21E‐04	21	0.86	2.92E‐04	11333	0.88 (0.8‐0.98)	1.71E‐02	21	0.88	2.01E‐02	7266	0.84 (0.74‐0.95)	5.15E‐03	21	0.84	5.66E‐03	4.88E‐01

a IGAP variants determined in the publication Lambert et al.^21^

b SNPs rs4147929 and rs9331896 were filtered out of the original analysis due to the QC procedures in Boehme et al.^27^ They were evaluated separately for the joint analysis.

c Chromosome, position, alleles from the February 2009 (GRCh37/hg19) build.

d Nearest gene(s) located within ±100 kb of the IGAP SNP.

e MAF calculated in PLINK derived from the unstratified genotypes.

f Unstratified contains all subjects from the 21 ADGC cohorts used for analysis. The H2 tagging variant rs8070723 was used to stratify study participants into H2 non‐carriers (H1H1) and H2 carriers (H1H2+H2H2).

g Joint analysis performed with logistic regression in PLINK using as covariates age, sex, cohort, and PC1‐3. Rs4147929 is missing in the GSK cohort. N, number of subjects analyzed; OR, odds ratio for AD risk for the minor allele; 95% CI, 95% confidence interval, *P*‐value.

h Meta‐analysis performed with logistic regression analysis in PLINK using the random effects model, and using the covariates age, sex, and PC1‐3. Meta‐analysis could not be performed for rs4147929 and rs9331896. N, number of cohorts analyzed; OR, odds ratio, *P*‐value.

i Epistasis analyses performed in R. All cohorts were combined and stratified into two groups by the H2 tagging variant rs8070723: H2 non‐carriers (H1H1) and H2 carriers (H1H2+H2H2). Results from the carrier model are shown.

### 3.2 Genome‐wide *MAPT* haplotype–specific AD risk association analysis

**FIGURE 1:**
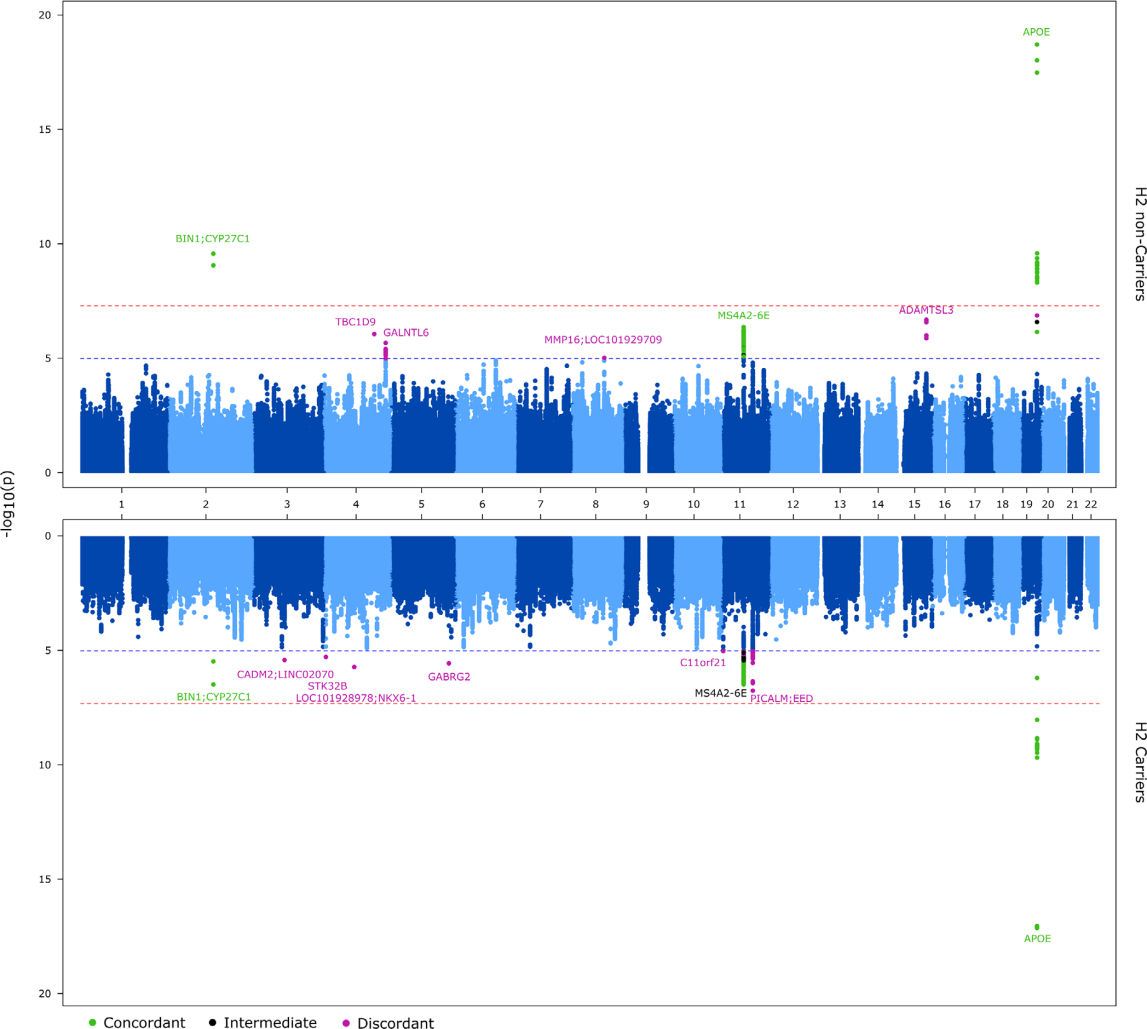
Miami plot of *MAPT* haplotype–stratified association results: *P* values from the joint association analyses are shown. *APOE* was not included as a covariate in these analyses. Top: H2 non‐carriers. Bottom: H2 carriers. The threshold for genome‐wide significance (*P* < 5E10‐8) is indicated by the red line and the threshold for trending significance (*p* < 1E‐5) is indicated by the blue line. Loci with *P* < 1E‐05 are annotated as follows: dark green, concordant (*P* < 1E‐05 in both data sets with epistatic *P* > 0.05); dark purple, discordant (*P* < 1E‐05 in one data set only, with epistatic *P* < 0.05); black, intermediate (*P* < 1E‐05 in one data set only with epistatic *P* > 0.05)

To identify any additional AD risk variants with *MAPT* haplotype–specific association, we evaluated the genome‐wide results for the unstratified, H2 non‐carrier and H2 carrier groups (Figure 1). We tested for significance of *MAPT* haplotype–specific associations by genome‐wide epistasis analysis with rs8070723 (Table 2). We defined loci with *MAPT* haplotype–specific AD risk associations as being discordant. To be classified as discordant, the following criteria had to be met: Discordant locus (1) has AD risk association *P* value of < 1E‐05 in one of the stratified analysis, but statistically insignificant in the other one (*P* > 5E‐02); (2) has nominally significant epistasis interaction with rs8070723 (*P* < 0.05).

**TABLE 2:** MAPT‐haplotype specific associations

						Unstratified^d^						H2 non‐carriers^d^						H2 carriers^d^						
SNP^a^	CHR^b^	Position^b^	Closest gene^c^	Major/Ref allele^b^	Minor/Alt allele^b^	N_j_ ^e^	OR_J_ (95% CI)^e^	P_j_ ^e^	N_m_ ^f^	OR_m_ ^f^	P_m_ ^f^	N_j_ ^e^	OR_J_ (95% CI)^e^	P_j_ ^e^	N_m_ ^f^	OR_m_ ^f^	P_m_ ^f^	N_j_ ^e^	OR_J_ (95% CI)^e^	P_j_ ^e^	N_m_ ^f^	OR_m_ ^f^	P_m_ ^f^	Epistatis P^g^
Discordant loci with *P* ≤ 1E‐5 in the H2 non‐carriers^a.1^																								
rs4555724	4	141658697	TBC1D9	T	C	18797	0.91 (0.87‐0.95)	3.64E‐05	21	0.92	3.19E‐03	11454	0.87 (0.82‐0.92)	8.64E‐07	21	0.87	1.11E‐05	7343	0.98 (0.92‐1.06)	6.60E‐01	21	0.99	6.95E‐01	6.78E‐03
rs28705797	4	173902942	GALNTL6	A	C	18759	1.13 (1.06‐1.2)	1.12E‐04	21	1.13	1.97E‐04	11424	1.21 (1.12‐1.32)	2.10E‐06	21	1.21	4.73E‐06	7335	1.02 (0.92‐1.13)	6.96E‐01	21	1.00	9.37E‐01	7.96E‐03
rs1685555	8	89483894	MMP16;LOC101929709	A	G	18637	0.79 (0.69‐0.9)	5.13E‐04	21	0.79	1.08E‐02	11371	0.68 (0.57‐0.81)	9.49E‐06	20	0.67	3.08E‐04	7266	1 (0.81‐1.24)	9.79E‐01	20	1.02	8.78E‐01	4.98E‐03
rs4354897	15	84644971	ADAMTSL3	T	C	18707	0.91 (0.87‐0.96)	2.90E‐04	21	0.91	5.72E‐03	11397	0.84 (0.79‐0.9)	2.04E‐07	21	0.84	1.22E‐06	7310	1.03 (0.95‐1.12)	4.87E‐01	21	1.02	7.13E‐01	1.22E‐04
rs11665676	19	45378719	NECTIN2;TOMM40:APOE	C	T	18622	0.77 (0.7‐0.85)	6.16E‐07	21	0.76	2.40E‐05	11355	0.7 (0.62‐0.8)	1.33E‐07	21	0.69	9.76E‐07	7267	0.89 (0.75‐1.05)	1.63E‐01	21	0.89	1.70E‐01	3.45E‐02
Discordant loci with *P* ≤ 1E‐5 in the H2 carriers^a.2^																								
rs7356060	3	86238011	CADM2;LINC02070	A	T	18424	1.13 (1.05‐1.21)	4.39E‐04	21	1.12	7.63E‐04	11231	1.04 (0.95‐1.13)	4.28E‐01	21	1.03	5.77E‐01	7193	1.29 (1.16‐1.43)	3.99E‐06	21	1.29	7.64E‐06	1.67E‐03
rs348732	4	85250583	LOC101928978;NKX6‐1	C	A	18605	0.91 (0.88‐0.96)	5.82E‐05	21	0.91	5.32E‐05	11342	0.96 (0.91‐1.02)	1.47E‐01	21	0.96	1.46E‐01	7263	0.84 (0.79‐0.91)	1.99E‐06	21	0.83	4.49E‐07	6.17E‐03
rs1838973	4	5157262	STK32B	G	A	18586	1.26 (1.14‐1.4)	1.40E‐05	21	1.26	2.19E‐05	11332	1.14 (1‐1.31)	5.43E‐02	21	1.14	6.95E‐02	7254	1.46 (1.24‐1.72)	5.46E‐06	20	1.56	1.27E‐05	2.28E‐02
rs55712126	5	161528378	GABRG2	T	G	18703	1.12 (0.99‐1.26)	6.85E‐02	21	1.09	1.66E‐01	11412	0.9 (0.78‐1.05)	1.85E‐01	20	0.89	1.36E‐01	7291	1.62 (1.33‐1.99)	2.88E‐06	21	1.60	9.85E‐06	3.91E‐06
rs77007065	11	2319730	C11orf21	G	A	18743	0.93 (0.84‐1.03)	1.79E‐01	21	0.94	3.07E‐01	11426	1.12 (0.99‐1.28)	7.88E‐02	21	1.12	8.86E‐02	7317	0.68 (0.58‐0.81)	9.78E‐06	20	0.70	8.35E‐05	5.62E‐06
rs140869727	11	85751041	PICALM;EED	G	A	18516	1.11 (1.06‐1.17)	5.00E‐05	21	1.12	3.79E‐04	11283	1.05 (0.98‐1.12)	1.57E‐01	21	1.05	3.05E‐01	7233	1.22 (1.12‐1.33)	3.00E‐06	21	1.23	2.38E‐06	4.51E‐03

This table shows the loci with AD risk associations of *P* ≤1E‐5 in only H2 non‐carriers (a.1) or H2 carriers (a.2). The H2 tagging variant rs8070723 was used to stratify study participants into H2 non‐carriers (H1H1) and H2 carriers (H1H2+H2H2). The table shows the top loci from the LD‐based clumping implemented in PLINK using an r2 threshold of 0.2 within 1000kb.

a.1 Loci with *P* ≤ 1E‐5 in H2 non‐carriers and *P* ≥ 0.05 in the in H2 carriers.

a.2 Loci with *P* ≤ 1E‐5 in H2 carriers and *P* ≥ 0.05 in the in H2 non‐carriers. Rs140869727 at the PICALM locus was clumped with rs17159904, but rs17159904 did not meet the *P* ≥ 0.05 threshold in the H2 non‐carriers. Therefore, the result from the former is shown.

b Chromosome, position, alleles from the February 2009 (GRCh37/hg19) build.

c Nearest gene(s) located within ±100 kb of the top SNP.

d Unstratified contains all subjects from the 21 ADGC cohorts used for analysis.

e Joint analysis performed with logistic regression in PLINK using as covariates age, sex, cohort and PC1‐3. N, number of subjects analyzed; OR, odds ratio; 95% CI, 95% confidence interval, P‐value.

f Meta‐analysis of logistic regression analysis in PLINK using the random effects model, and using the covariates age, sex and PC1‐3. N, number of cohorts analyzed; OR, odds ratio, P‐value.

g Epistasis analyses performed in R. All cohorts were combined and stratified into two groups by the H2 tagging variant rs8070723: H2 non‐carriers (H1H1) and H2 carriers (H1H2+H2H2). Results from the carrier model are shown.

We identified five loci in the H2 non‐carriers and six in the H2 carriers with discordant *MAPT* haplotype–specific AD risk associations (Figure 1, Table 2). These loci (nearest genes at loci) are as follows: In the H2 non‐carriers: chr4 (*TBC1D9*), chr4 (*GALNTL6*), chr8 (*MMP16;LOC101929709*), chr15 (*ADAMTSL3*), and chr19 (*NECTIN2;TOMM40;APOE*); and in the H2 carriers: chr3 (*CADM2;LINC02070*), chr4 (*STK32B*), chr 4 (*LOC101928978;NKX6‐1*), chr5 (*GABRG2*), chr11 (*C11orf21*), chr11 (*PICALM;EED*). None of these loci reached genome‐wide significance, although they had a stronger association in their relevant *MAPT* haplotype–stratified groups than in the combined unstratified group, despite the smaller sample size of the former. Forest plots of the discordant loci and their meta‐analysis results are shown in Figure S3.

**FIGURE 2:**
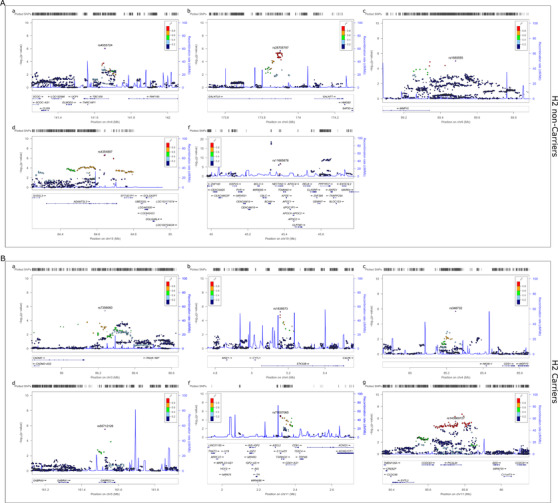
Regional association plots of discordant *MAPT* haplotype–stratified association results: The figures are shown for the 11 loci depicted in Table 2 and reflect the results of haplotype‐stratified joint association analyses without the *APOE* covariate. Discordant loci results with significance in the (A) H2 non‐carriers or (B) H2 carriers.

We checked the regional association plots of the discordant loci to determine whether any of them represented known IGAP AD risk loci (Figure 2). All but two of the discordant loci are novel, which is not surprising because the most significant associations detected by IGAP are likely to be enriched for concordant loci. The two discordant loci that are also known AD risk loci are *NECTIN2;TOMM40;APOE* and *PICALM;EED*, which have differentially greater significance in the *MAPT* H2 non‐carriers and H2 carriers, respectively. We further evaluated these two loci to determine the extent to which the discordant associations are influenced by the known index variants.

For the *NECTIN2;TOMM40;APOE* locus, we determined that the minor T allele of rs11665676 is more enriched in *APOE* ε4–negative than in *APOE* ε4–positive participants, with allele frequencies of 0.06 versus 0.03, respectively. The frequency of rs11665676‐T in participants with the *APOE* ε2/ε2; ε2/ε3; ε2/ε4; ε3/ε3; ε3/ε4; and ε4/ε4 backgrounds is 0; 0.037; 0.006; 0.065; 0.035; and 0.006, respectively, which demonstrates the enrichment of this allele, particularly in *APOE* ε3. When we repeated the analysis adjusting for *APOE* ε4 dosage, the AD risk association of rs11665676 in *MAPT* H2 non‐carriers was abolished (*APOE*‐unadjusted OR = 0.7 and *P* = 1.33E‐07; *APOE*‐adjusted OR = 0.93 and *P* = 0.28), which is not surprising given the strong linkage disequilibrium (LD) of this variant with those that define *APOE* ε2/ε3/ε4 (rs429358 and rs7412). *APOE* ε4 dosage association with AD risk did not reveal differences between the *MAPT* H2 non‐carriers (OR = 1.35, *P* = 3.28E‐264) and H2 carriers (OR = 1.42, *P* = 2.80E‐190). The 95% CI for *APOE* ε4 AD risk ORs were overlapping between these two stratified groups, and there was no evidence of epistasis interaction between *APOE* ε4 dose and *MAPT* H1/H2 haplotype. Collectively, our findings suggest that although there are no discordant associations for *APOE* ε4 dose per se based on *MAPT* H1/H2 status, rs11665676‐T may be tagging a subtype of *APOE*, which confers greater protection in *MAPT* H2 non‐carriers.

The *PICALM* locus index IGAP SNP rs10792832 did not have any evidence of differential *MAPT* haplotype–stratified association (Table 1, epistasis *P* value > 0.05). In contrast, the discordant variant rs140869727 that resides in an intron of *PICALM* has epistasis (*P* = 4.51E‐03) with AD risk association in the *MAPT* H2 carriers (OR = 1.22, *P* = 3.0E‐06, Table 2). The linkage disequilibrium *r* ^2^ value for these SNPs is 0.15 in the unstratified and both stratified cohorts, with *D*' = 0.99. These results support a model where the rarer and discordant rs140869727‐A may be tagging a *PICALM* variant, which confers a greater risk of AD in *MAPT* H2 carriers.

Of the discordant loci with significance in the H2 non‐carriers, the four novel ones had essentially no overlap in their 95% CIs with the H2‐carrier results (Table 2). The level of significance for joint analyses in the H2 non‐carriers ranged between *P* = 2.04E‐7 (*ADAMTSL3*) and *P* = 9.49E‐6 (*MMP16;LOC101929709*). For these discordant variants that are significant in the H2 non‐carriers, there was no evidence of association in the H2 carriers (ORs ≈1 and *P* = 0.5‐1.0). Similarly, the five discordant novel loci with significance in the H2 carriers had *P* = 1.99E‐6 (*LOC101928978;NKX6‐1*) to *P* = 9.78E‐6 (*C11orf21*), whereas in the H2 non‐carriers, these variants had ORs at ≈1 with essentially non‐overlapping 95% CIs and *P* = 0.054 to 0.4.

By definition, all discordant loci had nominally significant epistasis *P* values, although none reached genome‐wide significance (Table 2). Considering the 21 IGAP and 11 discordant loci evaluated, and applying a study‐wide epistasis *P* ‐value of 1.52E‐3 (Bonferroni *P* = 0.05/33), there was one discordant SNP with *MAPT* H2 non‐carrier–specific association and two discordant SNPs in the *MAPT* H2‐carrier group. The SNP with the smallest epistasis *P* value and *MAPT* H2 non‐carrier–specific association is rs4354897 on chromosome 15 (Table 2), an intronic variant within *ADAMTSL3* (Figure 2). Among the discordant loci, this is the second most significant variant (*P* = 2.04E‐07) after the chromosome 19 *APOE* locus variant. The minor allele of *ADAMTSL3* rs4354897 is associated with a lower risk of AD (OR = 0.84) in *MAPT* H2 non‐carriers.

The two *MAPT* H2 carrier–specific variants with study‐wide significant epistasis were rs55712126 on chromosome 5, an intronic variant in *GABRG2*; and rs77007065 on chromosome 11, which is intronic for *C11orf21* and also 2 kb upstream of *TSPAN32* (Figure 2). *GABRG2* rs55712126‐G and *C11orf21* rs77007065‐A are associated with higher (OR = 1.62, *P* = 2.88E‐06) and lower AD risk (OR = 0.68, *P* = 9.78E‐06), respectively, in *MAPT* H2 carriers (Table 2).

To determine the joint effect of the discordant SNPs and *MAPT* haplotypes on AD risk, we performed a bivariate analysis (Table S2). The *MAPT* H2 non‐carriers with the SNP major homozygote genotypes were designated as the reference. We tested the AD risk association of each SNP genotype in the *MAPT* H2‐carrier or H2 non‐carrier background against this reference. The bivariate analysis results are consistent with their corresponding *MAPT* haplotype–specific associations and depict the joint effect of each SNP genotype and the *MAPT* haplotype on AD risk.

### 3.3 Brain expression analyses of *MAPT*‐stratified AD risk association loci genes

We characterized the brain expression patterns of the genes at the discordant *MAPT*‐stratified association loci (Table 2) using the temporal cortex (TCX) RNAseq transcriptome data from Mayo Clinic.^22^, ^23^, ^32^ Of the 17 genes at the 11 discordant loci, 12 were present in this data set (Table S3). We evaluated these genes for differential expression (DE) between neuropathologic AD and control TCX RNAseq data. In addition, we determined the brain gene co‐expression networks,^34^ which harbor these genes and annotated these networks for their enriched gene ontology (GO) biological processes^35^ and brain cell types, as described previously.^23^, ^32^ Eight of the 12 genes evaluated had significantly different expression in AD versus control TCX (Table S3). The genes with transcriptome‐wide significant differential expression (q<0.05) were *GALNTL6, TBC1D9, TOMM40, APOE, PVRL2, ADAMTSL3, GABRG2*, and *PICALM*, with *q* values ranging between 1.99E‐02 and 3.29E‐06.

Of interest, six of these genes reside in a co‐expression network module (TCX1) that is enriched for both neuronal cell types and “synaptic transmission” GO process (GO:0007268). The “synaptic transmission” module itself is also associated with AD (*P* = 4.70E‐03). Four of the (*GALNTL6, TOMM40, TBC1D9, MMP16*) “synaptic transmission” module genes had *MAPT* H2 non‐carrier, and the other two (*CADM2, GABRG2*) had H2 carrier–specific AD risk association (Table 2). The “synaptic transmission” module and all but one discordant gene in this module were lower in AD TCX, as would be expected from neuronal loss observed in AD brains in this region. The discordant loci genes *GALNTL6, TBC1D9*, and *GABRG2* had high module membership levels >0.80, suggesting strong correlations with the rest of the network. Notably, *MAPT* also resides in the ``synaptic transmission” module: TCX1. Of the IGAP loci genes, *PTK2B, EPDR1*, and *CELF1* also reside within TCX1.

Of the other differentially expressed genes, two were from modules that had cell type enrichment: *NECTIN2* (*PVRL2*) belonged to the module enriched for “defense response” (GO:0006952) and microglia. Both the *NECTIN2* (*PVRL2*) gene (differential expression = DE *q* = 9.93E‐04) and its module (DE *P* = 4.19E‐06) had significant differential expression in the AD versus control brains (Table S3). The other was *APOE* (DE *q* = 9.42E‐04), which resided in the module (DE *P* = 1.12E‐04) enriched for “carboxylic acid catabolic process” (GO:0046395), and both astrocytes and endothelia. These genes reside at the same chromosome 19 locus. Both of these modules and genes were higher in the AD TCX, which may again be expected based on microglial and astrocytic population increases observed in brain regions affected with AD neuropathology. Finally, two genes, *ADAMTSL3* (DE q = 1.53E‐03) and *PICALM* (DE q = 7.72E‐03), which reside at *MAPT* H2 non‐carrier and H2 carrier–specific loci, respectively, are both significantly higher in AD TCX and belong to modules enriched for “regulation of transcription, DNA‐templated” (GO:0006355) (DE *P* = 3.71E‐02).

We performed the same analyses also for the known AD risk loci genes (Table S3). Eight of the 17 IGAP loci genes with brain expression data had significant differential expression, both at the gene (*q* < 0.05) and module levels (*P* < 0.05). Two modules enriched for “immune response” (GO:0006955) and “synaptic transmission” (GO:0007268) genes had the highest number of IGAP risk loci genes. Three genes (*HLA‐DRB1, INPP5D, MS4A6A*) were in the microglial gene–enriched “immune response” module, as we have shown previously^36^; and three others (*CELF1, EPDR1, PTK2B*) were in the neuronal gene enriched “synaptic transmission” module. We noted that there were IGAP risk loci genes within oligodendrocyte (*BIN1, ZCWPW1*), astrocyte/endothelia (*CLU, FERMT2*), and endothelia gene–enriched modules (*CASS4*). In summary, half of the discordant *MAPT*‐stratified loci genes were from neuronal modules, whereas the IGAP AD risk loci genes had similar representation across network modules that were enriched for any of the five brain cell types.

To determine whether any of the *MAPT* haplotype‐specific AD risk SNPs influenced brain expression levels of *MAPT* or the “synaptic transmission” co‐expression module TCX1, which also harbors *MAPT*, we performed expression quantitative trait loci (eQTL) and module QTL (modQTL) analyses, respectively, as described previously.^14^, ^32^, ^37^, ^38^ None of the *MAPT* haplotype–specific AD risk SNPs had significant associations with temporal cortex *MAPT* levels or the “synaptic transmission” module eigengene (data not shown). We conclude that these *MAPT* haplotype‐specific loci are not likely to confer AD risk through their influence on brain gene expression of *MAPT* or synaptic transmission network genes.

## 4 DISCUSSION

Despite significant progress in identifying genetic risk factors and the increased understanding in Alzheimer's disease (AD) etiology, the ability to develop effective preventions or cures continues to remain elusive. Novel approaches to analyzing available multiscale genomic and phenotypic data will provide further insights into the complexity of AD and provide mechanisms to foster the development of precision medicine.

In this study we sought to evaluate available genome data by performing a stratified analytic approach. Stratified methods based on sex^39^, ^40^, ^41^ and *APOE* ^19^ have been reported previously and have shown background‐dependent associations with AD. Due to the implication of MAPT in both AD neuropathology^1^, ^7^, ^20^ and risk,^14^, ^15^, ^18^ we performed *MAPT* haplotype–stratified association analyses in the genotype data from the ADGC to test the hypothesis that AD risk variants may exhibit *MAPT* haplotype‐dependent association. We tested previously identified AD risk loci^21^ to determine whether they have differential associations in a *MAPT* haplotype context–dependent manner. We also extended this analysis genome‐wide to determine if this approach may identify novel AD risk variants.

We found that the index AD risk variants reported previously had similar directions of associations in both the *MAPT* H2 non‐carrier and H2 carrier analyses. Epistasis analysis with these and the *MAPT* H1/H2 haplotype tagging variants revealed no evidence of differential association (*P* > 0.05) for all but two AD risk loci. Even though *CD2AP*‐rs10948363 and *ZCWPW1/PILRB*‐rs1476679 had nominally significant epistasis (*P* = 0.035 and 0.022, respectively), the estimated effects of these variants were largely overlapping in the *MAPT* H2 carriers and non‐carriers. These findings are not surprising given that the loci that rise to significance in the overall GWAS are likely to have a more consistent effect across stratified groups.

In contrast, stratified analysis may uncover novel loci with group‐specific associations that may be missed in the combined cohort. Although we did not identify any *MAPT* haplotype–specific associations at genome‐wide significance in this study, we uncovered 11 discordant loci that had association at *P* < 1E–05 in one stratum (five in *MAPT* H2 non‐carriers and six in *MAPT* H2‐carriers), no association (*P* > 0.05) in the other stratum and evidence of epistasis (*P* < 0.05) with the *MAPT* H1/H2 tagging variant rs8070723. The most significant *MAPT*–haplotype–specific association was observed for chromosome 19 variant rs11665676 at the *NECTIN2;TOMM40;APOE* locus. The minor T allele of this variant was associated with a lower AD risk (OR = 0.7, *P* = 1.33E‐07) only in the *MAPT* H2 non‐carriers (ie, those with *MAPT* H1/H1 haplotype). It is important to note that although there was no evidence of *MAPT* haplotype–specific associations for *APOE* ε4 dose in our study per se, rs11665676‐T is enriched in *APOE* ε3 carriers.

These findings suggest the following model: In the presence of the strong effect conferred by *APOE* ε4, the presence of *MAPT* H1 versus H2 haplotype does not make a significant difference with respect to AD risk. Consequently, there is no *MAPT* haplotype–specific associations for *APOE* ε4 dose. However, rs11665676‐T, which is enriched in *APOE* ε3 carriers, may be marking a variant of *APOE* that confers greater protection in those who are *MAPT* H2 non‐carriers. We and others previously showed that *MAPT* H2 haplotype is associated with a lower risk of AD.^14^, ^15^, ^18^ The preferential protection of rs11665676‐T in *MAPT* H2 non‐carriers may be due to the fact that in the presence of the protective *MAPT* H2 haplotype, any further protection conferred by this variant may be negligible. This may explain the lack of association of rs11665676‐T with lower AD risk in *MAPT* H2‐carriers.

The discordant rs11665676 variant resides within an intron of *NECTIN2* (aka *PVRL2*), which is within a LD region with *BCAM* and in proximity to the *TOMM40‐APOE‐APOC1* locus.^42^ It has been shown previously that the LD structure of the polymorphisms across these five genes displayed heterogeneity between AD and control individuals, suggesting that the genes within this region in addition to *APOE* may play a role in AD risk.^42^, ^43^ Indeed, a highly polymorphic variant of *TOMM40* (poly‐T variant) was found to associate with AD risk and its endophenotypes independent of *APOE* in some studies.^44^ Given the complexity of this region on chromosome 19, including LD across multiple genes, plentiful polymorphisms, and the strong *APOE* ε2/ε3/ε4 effect on AD risk, alternative approaches focused on haplotype analysis of this region are proposed to uncover novel variants that influence AD independent of *APOE*.^45^ Our analysis of stratifying samples according to specific genotypic/haplotypic backgrounds provides another approach in the discovery of polymorphisms that may influence AD risk under a specific genomic context. Our approach identified a polymorphism in *NECTIN2* (*PVRL2*), which is enriched in *APOE* ε3 carriers and which has differential protective association in *MAPT* H2 non‐carriers. This finding suggests a biological link between *NECTIN2* and/or *APOE* with *MAPT*.

In a previous *APOE*‐stratified analysis,^19^ a variant in the *MAPT* region, rs2732703‐G, which is more common in H2 carriers, was found to confer greater protection from AD in *APOE* ε4 negative individuals. This finding is different and independent of our report, and suggests that variability at the *MAPT* locus influences *APOE* association with AD risk, whereas our results indicate that variability at the *APOE* locus has distinct AD risk association on different *MAPT* haplotype backgrounds. Both findings support the notion of heterogeneity at both *APOE* and *MAPT* haplotypic regions, which may modify AD risk associations depending on the combinations of variants harbored. Understanding the full set of functional variants at these important loci, their genetic/biological interactions, and their collective effects on AD risk and its endophenotypes is necessary to successfully practice precision medicine in the future.

Whether the *NECTIN2* (aka *PVRL2*) rs11665676 variant signifies association with this gene per se or marks another variant within *APOE* remains to be established. *NECTIN2* (nectin, cell adhesion molecule 2), also known as poliovirus receptor‐related 2 (and formerly as herpesvirus entry mediator B, HVEB), encodes a plasma membrane glycoprotein that has been implicated in a multitude of central nervous system (CNS) functions.^43^ NECTIN2 is involved in adherens junction, which is important to maintain blood‐brain barrier and to prevent the spread of viral infections. In our brain expression data^22^, ^23^, ^32^ analyzed herein, we determined *NECTIN2* to be significantly elevated in AD TCX, and to reside in a co‐expression module enriched for “defense response” GO biological process and microglia‐enriched genes. These findings support a role for this gene in innate immune pathways. Our findings along with prior association of another *NECTIN2* variant (rs6859) with AD risk in African Americans independent of *APOE*,^46^ merit further evaluation of this gene as a plausible AD gene.

In addition to the *NECTIN2* variant at the *APOE* locus, *MAPT*‐stratified analysis revealed one other discordant association in a known AD risk locus, which was *PICALM* intronic SNP rs140869727 that revealed increased risk in *MAPT* H2‐carriers. The minor A allele of rs140869727 has frequency (MAF) of 0.17 and is rarer than the *PICALM* locus index IGAP SNP rs10792832, which has a MAF of 0.36. The latter did not have differential *MAPT* haplotype–stratified association, whereas rs140869727 had epistasis (*P* = 4.51E‐03). We concluded that the discordant rarer SNP may be tagging a *PICALM* variant, which confers greater risk of AD in *MAPT* H2 carriers. PICALM was found to associate with both 3R and 4R tau inclusions in AD and primary tauopathies, and soluble PICALM levels were inversely correlated with phosphotau,^47^ suggesting a biological link between this protein involved in clathrin‐mediated endocytosis and tau.

We identified nine discordant loci that were not previously identified in AD risk GWAS, including the largest recent studies.^48^, ^49^ The four novel H2 non‐carrier–specific associations were near *TBC1D9* (chr4), *GALNTL6* (chr 4), *MMP16;LOC101929709* (chr8), and *ADAMTSL3* (chr15). Of these, *ADAMTSL3* locus had the strongest AD risk association (*P* = 2.04E‐7), where the minor allele of intronic SNP rs4354897 conferred protection (OR = 0.84, CI = 0.79 to 0.9), only in *MAPT* H2 non‐carriers, but not in H2 carriers (epistasis *P* = 1.22E–04). *ADAMTSL3* encodes a glycoprotein that localizes to the extracellular matrix, belongs to a family of metalloproteases, and is proposed to be a candidate gene for schizophrenia, with proposed function in synaptogenesis.^50^ Of interest, another H2 non‐carrier–specific association locus resides near a different matrix metalloproteinase encoding gene, *MMP16*. Matrix metalloproteases have been implicated in AD and other neurodegenerative diseases through their roles in Aß degradation, inflammatory processes, and processing of neurodegenerative proteins including tau.^51^ Given this, metalloproteases have been proposed as potential therapeutic targets in AD and other neurodegenerative diseases. The other two genes at *MAPT* H2 non‐carrier–specific AD risk loci have been identified previously in vascular and/or neuropsychiatric genetic studies. *G*ALNTL6 has been associated with lipid metabolism,^52^ body mass index,^53^ and hypertension. In addition, a separate SNP in *GALNTL6* was associated with AD at age of onset, although it lost its significance after correcting for the *APOE*.^54^ *TBC1D9* is a brain‐expressed gene encoding a protein with Rab3A‐GAP activity. There are no reports linking this gene to AD to date. Recently, a de novo and potentially pathogenic *TBC1D9* missense variant was identified in sporadic Attention‐Deficit/ Hyperactivity Disorder (ADHD).^55^

Five novel loci showed AD risk association only in the H2 carriers, namely, *CADM2;LINC02070* on chromosome 3, *STK32B* on chromosome 4, *LOC101928978;NKX6‐1* on chromosome 4, *GABRG2* on chromosome 5, and *C11orf21* on chromosome 11. Of these, *GABRG2* locus has the strongest AD risk association (*P* = 2.88E‐06) and evidence of epistasis with *MAPT* H1/H2 locus (3.91E‐06). *GABRG2* encodes the γ2 subunit of the pentameric γ‐aminobutyric acid receptor A (GABA_A_) ligand‐gated ion channels that bind the major inhibitory neurotransmitter in mammalian brains, GABA. Previously, missense, nonsense, frameshift, splice‐site, and deletion mutations within *GABRG2* were associated with simple febrile seizures and genetic epilepsy syndromes through different mechanisms leading to reduced channel levels and/or function.^56^ *GABRG2* levels were found to be reduced in iPSC‐derived neurons and brains from *MAPT* p.R406W carriers, mouse models of tauopathy,^57^ and in the Mayo Clinic brain RNAseq data^22^ from patients with the primary tauopathy PSP compared with controls, in both TCX and cerebellum. In our study, we also evaluated the Mayo Clinic brain RNAseq data and determined lower levels of *GABRG2* in TCX (q = 1.99E‐02), but not in the cerebellum (data not shown) in AD compared with controls. Collectively, these findings suggest that tauopathies could lead to lower expression of the inhibitory channel proteins, including *GABRG2*, possibly through loss of these neurons in affected brain areas. This could in turn lead to excitatory/inhibitory imbalance, culminating in enhanced Aß production and ultimately further neuronal loss.^58^ Our findings suggest that *GABRG2* variants increase AD risk preferentially in *MAPT* H2 carriers, who are expected to have lower brain *MAPT* levels and greater protection against AD.^14^ Hence, risk conferred by other pathways, such as disruption of GABAergic signaling, may be more important for and detectable in this *lower MAPT risk* group.

The intronic variant rs7356060 that discordantly confers risk in *MAPT* H2 carriers (OR = 1.29, CI = 1.16 to 1.43, *P* = 3.99E‐06) marks another interesting candidate *CADM2*, which was also identified as a candidate gene in a GWAS of cognitive function, specifically executive function and processing speed.^59^ *CADM2* encodes cell adhesion molecule 2 and is also known as synaptic cell adhesion molecule 2 (*SYNCAM2*) and nectin‐like protein 3 (*NECL3*). That the *MAPT*‐stratified analysis led to the discovery of a nectin (*NECTIN2* on chromosome 19) and a nectin‐like protein (*CADM2* = *NECL3* = *SYNACM2*) as candidates is noteworthy. *CADM2* was also identified as a locus for habitual physical activity, along with *APOE*,^60^ and was also suggested as a gene that may link obesity with psychiatric traits.^61^

The three other candidate genes at the AD risk loci identified in *MAPT* H2‐carriers—*C11orf21*, *STK32B*, and *NKX6‐1*—were also implicated in CNS diseases or function. *C11orf21* has an intronic variant rs77007065‐A, which confers AD protection in *MAPT* H2 carriers (0.68, CI = 0.58 to 0.81, *P* = 9.78E‐06) and is one of the most discordant SNPs (epistasis *P* = 5.62E‐06). This variant is also upstream of *TSPAN32*, which together with *C11orf21* resides in a region of differential methylation in autistic brain samples.^62^ *STK32B* is a serine/threonine kinase and resides at a locus previously identified in a GWAS for essential tremor.^63^ The promoter region of this gene is differentially methylated in blood samples from adolescents with generalized anxiety disorder.^64^ Finally, *NKX6‐1*, which is a transcription factor, was found to be involved in midbrain dopaminergic neuron differentiation,^65^ in addition to its role in the differentiation of pancreatic ß islet cells.^66^ Whether these are the genes that harbor functional variants that influence AD risk in a *MAPT* haplotype–dependent manner and their biological interaction with tau‐related pathways remains to be established.

In our study, we also performed a systematic evaluation of all of the candidate genes at the discordant AD risk loci for their expression in AD versus control temporal cortex (TCX),^22^, ^23^, ^32^ their membership in brain gene co‐expression networks identified in these samples, and annotation of these networks for their enriched biological processes and CNS cell types. For these analyses, we utilized the Mayo Clinic Brain RNAseq data generated by our group, and implemented approaches as previously described.^22^, ^23^, ^32^ We also analyzed the candidate genes at the known IGAP AD risk loci^21^ in the same fashion. Eight of 12 discordant loci genes and eight of 17 IGAP loci genes were differentially expressed in AD versus control TCX with transcriptome‐wide significance (*q* < 0.05). Half of the discordant loci genes (*GALNTL6, TOMM40, TBC1D9, GABRG2, MMP16, CADM2*) were members of the co‐expression network that was enriched in “synaptic transmission” GO biological process. This network had also a significantly higher representation of neuron‐enriched genes. In comparison to the discordant loci genes, the known IGAP AD risk loci genes had a lower representation of “synaptic transmission” membership, with 3 (*PTK2B, EPDR1, CELF1*) of 17 genes that were assessed in the transcriptome data. The published IGAP loci genes had membership within a variety of networks with broader enrichment of GO processes and cell types. These included “axon ensheathment”/oligodendrocyte (*BIN1, ZCWPW1*); “immune response”/microglia (*HLA‐DRB1, INPP5D, MS4A6A*); “carboxylic acid catabolic process”/astrocyte and endothelia (*CLU; FERMT2*); and “vasculature development”/endothelia (*CASS4*). Neither GWAS associations nor co‐expression network and differential gene expression analyses per se definitively identifies the disease risk genes. Nevertheless, the concurrent presence of GWAS candidate genes within networks that are enriched in processes known to be perturbed in the disease process (such as “immune response,” “synaptic transmission,” “axon ensheathment”) provides further strength for the candidacy of these genes and information about the pathways with which they are likely to be involved. The presence of half of the discordant loci in “synaptic transmission” networks suggests that the *MAPT* haplotype stratified approach may be preferentially identifying neuronal genes that are involved in this crucial process in a *MAPT* haplotype–dependent manner. This finding is congruent with known and proposed roles of tau in synaptic transmission or its disruption in AD.^67^ In comparison, the un‐stratified GWAS appears to uncover genes that pertain to a wider spectrum of pathways and cellular processes that may be due to the lack of the dependency on *MAPT* haplotype context.

Because the transcriptome data was obtained in bulk brain tissue in a region affected with AD neuropathology, the observed transcriptional differences between AD and controls may reflect cell population changes.^22^ Despite this caveat, we and others have successfully utilized bulk brain transcriptome data to identify transcriptional networks that associate with neurodegenerative diseases and their endophenotypes.^23^, ^32^, ^42^, ^43^, ^45^ Many of these networks are enriched in pathways and genes that have been implicated previously in these diseases through independent data including genetic associations.^32^, ^36^, ^42^ This suggests that integrative analysis of transcriptional networks and disease association data can provide cross‐validation for the genes. This approach also provides transcriptional context for the candidate genes discovered from disease GWAS as demonstrated here.

In summary, we performed a *MAPT* H1/H2 haplotype–stratified association in the ADGC GWAS data and identified 11 loci with evidence of association in one stratum (*P* < 1.0E‐05), no association in the other stratum (*P* > 0.05), and epistasis (*P* < 0.05) with the *MAPT* H1/H2 haplotype–tagging variant rs8070723. With the exception of a *NECTIN2* variant at the *NECTIN2;TOMM40;APOE* locus and a rare variant in *PICALM*, these are novel loci that have not been reported previously. Half of the candidate genes at these loci reside within a co‐expression network enriched in neuronal genes and implicated in “synaptic transmission.” These findings contrast with those from the known IGAP loci, where we did not find evidence of *MAPT* H1/H2 haplotype–stratified association and where the candidate genes are members of co‐expression networks that represent a broader range of cellular and biological process enrichment.

There are several limitations to our study. Notwithstanding their novelty, the *MAPT* H1/H2 haplotype–stratified association results should be interpreted with caution due to falling short of genome‐wide significance (*P* < 5.0E‐08), as they may represent false‐positive findings. It will be important to apply this approach in larger available GWAS data and seek confirmation. Given that *MAPT* H2 haplotype is rarer, our *MAPT* H2 carriers were smaller in size (*n* = 7360) than *MAPT* H2 non‐carriers (*n* = 11,481). This may explain the presence of two loci that approached genome‐wide significance in the *MAPT* H2 non‐carriers, whereas the strongest association remained at *P* = 1.99E‐06 in the *MAPT* H2 carriers. We also acknowledge that our *MAPT* H1/H2 haplotype definition was based on the tagging variant rs8070723 and that the H1 haplotype, which has considerable variation,^10^, ^11^ can be divided into additional sub‐haplotypes. Future studies utilizing whole genome sequencing (WGS) can enable more accurate assignment of haplotypes, although sub‐haplotypic stratification would require even greater sample sizes. We discovered that many of the candidate genes at the discordant AD risk loci are differentially expressed in AD TCX and reside in the “synaptic transmission” co‐expression network, which also harbors *MAPT*. Despite their intriguing biological implications, it is possible that these congruent genomic and transcriptomic findings are coincidental. Definitive determination of biological interactions between the discordant loci genes with *MAPT* requires detailed studies in model systems, which is beyond the scope of this work. Our findings provide testable hypotheses for such functional studies. Finally, our brain transcriptome data are driven from bulk tissue, where the gene expression findings may simply reflect cell population changes and where biologically important differential expression results in rarer cell types may be obscured. It will be important to evaluate brain cell–type specific expression patterns of the genes nominated in this study in the single‐nucleus and single‐cell transcriptome data from AD and control brains, once sizable data sets become available.

Our study represents an alternative approach in leveraging available GWAS data for discovery of loci and genes that may confer AD risk in a *MAPT* context–dependent manner. Integrative utilization of independent genomic and transcriptomic data provide cross‐validation for our findings. The candidate genes that emerge from this study should be evaluated for the presence of functional variants that may influence tau‐related outcomes in model systems or human cohorts. Emerging larger cohorts with multi‐omics data and generation of more complex model systems should enable these future studies.

## ADGC Affiliations

^1^Department of Neurology, Johns Hopkins University, Baltimore, Maryland, ^2^Department of Neurology, University of Michigan, Ann Arbor, Michigan, ^3^Geriatric Research, Education and Clinical Center (GRECC), VA Ann Arbor Healthcare System (VAAAHS), Ann Arbor, Michigan, ^4^Michigan Alzheimer Disease Center, Ann Arbor, Michigan, ^5^Department of Neurology, University of California Los Angeles, Los Angeles, California, ^6^Department of Psychiatry, University of Pennsylvania Perelman School of Medicine, Philadelphia, Pennsylvania, ^7^Geriatric Research, Education and Clinical Center (GRECC), University of Wisconsin, Madison, Wisconsin, ^8^Department of Medicine, University of Wisconsin, Madison, Wisconsin, ^9^Wisconsin Alzheimer's Institute, Madison, Wisconsin, ^10^Department of Medicine (Genetics Program), Boston University, Boston, Massachusetts, ^11^Department of Pharmacology and Neuroscience, University of North Texas Health Science Center, Fort Worth, Texas, ^12^Department of Human Genetics, University of Pittsburgh, Pittsburgh, Pennsylvania, ^13^Department of Neurological Sciences, Rush University Medical Center, Chicago, Illinois, ^14^Department of Behavioral Sciences, Rush University Medical Center, Chicago, Illinois, ^15^Civin Laboratory for Neuropathology, Banner Sun Health Research Institute, Phoenix, Arizona, ^16^Departments of Psychiatry, Neurology, and Psychology, University of Pittsburgh School of Medicine, Pittsburgh, Pennsylvania, ^17^The John P. Hussman Institute for Human Genomics, University of Miami, Miami, Florida, ^18^Dr. John T. Macdonald Foundation Department of Human Genetics, University of Miami, Miami, Florida, ^19^National Alzheimer's Coordinating Center, University of Washington, Seattle, Washington, ^20^Rush Alzheimer's Disease Center, Rush University Medical Center, Chicago, Illinois, ^21^Department of Pathology, Northwestern University Feinberg School of Medicine, Chicago, Illinois, ^22^Cognitive Neurology and Alzheimer's Disease Center, Northwestern University Feinberg School of Medicine, Chicago, Illinois, ^23^Department of Neurology, University of Washington, Seattle, Washington, ^24^VA Puget Sound Health Care System/GRECC, Seattle, Washington, ^25^Department of Epidemiology, Harvard School of Public Health, Boston, Massachusetts, ^26^Department of Psychiatry, Massachusetts General Hospital/Harvard Medical School, Boston, Massachusetts, ^27^Department of Neurology, Mayo Clinic, Rochester, Minnesota, ^28^Swedish Medical Center, Seattle, Washington, ^29^Department of Neurology, University of California San Francisco, San Francisco, California, ^30^Department of Medicine, Duke University, Durham, North Carolina, ^31^Department of Neuroscience, Mount Sinai School of Medicine, New York, New York, ^32^Department of Psychiatry, Mount Sinai School of Medicine, New York, New York, ^33^Departments of Genetics and Genomic Sciences, Mount Sinai School of Medicine, New York, New York, ^34^Department of Pathology and Immunology, Washington University, St. Louis, Missouri, ^35^Department of Pathology and Laboratory Medicine, University of Pennsylvania Perelman School of Medicine, Philadelphia, Pennsylvania, ^36^USF Health Byrd Alzheimer's Institute, University of South Florida, Tampa, Florida, ^37^Fred Hutchinson Cancer Research Center, Seattle, Washington, ^38^Department of Psychiatry and Behavioral Sciences, Miller School of Medicine, University of Miami, Miami, Florida, ^39^Department of Pathology, University of Alabama at Birmingham, Birmingham, Alabama, ^40^Department of Neurology, University of Southern California, Los Angeles, California, ^41^Department of Neurology, University of Alabama at Birmingham, Birmingham, Alabama, ^42^Neurogenomics Division, Translational Genomics Research Institute, Phoenix, Arizona, ^43^Department of Medicine, University of Washington, Seattle, Washington,^44^Department of Neurology, University of California Irvine, Irvine, California, ^45^Department of Psychiatry and Hope Center Program on Protein Aggregation and Neurodegeneration, Washington University School of Medicine, St. Louis, Missouri, ^46^Program in Translational NeuroPsychiatric Genomics, Institute for the Neurosciences, Department of Neurology & Psychiatry, Brigham and Women's Hospital and Harvard Medical School, Boston, Massachusetts, ^47^Program in Medical and Population Genetics, Broad Institute, Cambridge, Massachusetts, ^48^Department of Neurology, University of California Davis, Sacramento, California, ^49^University of Virginia School of Medicine, Charlottesville, Virginia, ^50^Institute for Memory Impairments and Neurological Disorders, University of California Irvine, Irvine, California, ^51^Wien Center for Alzheimer's Disease and Memory Disorders, Mount Sinai Medical Center, Miami Beach, Florida, ^52^Rush Institute for Healthy Aging, Department of Internal Medicine, Rush University Medical Center, Chicago, Illinois, ^53^Department of Medical and Molecular Genetics, Indiana University, Indianapolis, Indiana, ^54^Department of Neurology, Indiana University, Indianapolis, Indiana, ^55^Department of Psychiatry, New York University, New York, New York, ^56^C.S. Kubik Laboratory for Neuropathology, Massachusetts General Hospital, Charlestown, Massachusetts, ^57^Department of Neurosciences, University of California San Diego, La Jolla, California, ^58^Department of Psychiatry, University of Pittsburgh, Pittsburgh, Pennsylvania, ^59^Department of Pathology and Laboratory Medicine, Emory University, Atlanta, Georgia, ^60^Emory Alzheimer's Disease Center, Emory University, Atlanta, Georgia, ^61^Neurogenetics Program, University of California Los Angeles, Los Angeles, California, ^62^Department of Pathology and Laboratory Medicine, Indiana University, Indianapolis, Indiana, ^63^Department of Neurology, Emory University, Atlanta, Georgia, ^64^Division of Genetics, Department of Medicine and Partners Center for Personalized Genetic Medicine, Brigham and Women's Hospital and Harvard Medical School, Boston, Massachusetts, ^65^Department of Neurology, Massachusetts General Hospital/Harvard Medical School, Boston, Massachusetts, ^66^Center for Applied Genomics, Children's Hospital of Philadelphia, Philadelphia, Pennsylvania, ^67^Department of Pathology (Neuropathology), University of Pittsburgh, Pittsburgh, Pennsylvania, ^68^Institute of Neurology, University College London, Queen Square, London,^69^Sanders‐Brown Center on Aging, Department of Molecular and Biomedical Pharmacology, University of Kentucky, Lexington, Kentucky, ^70^Taub Institute on Alzheimer's Disease and the Aging Brain, Department of Neurology, Columbia University, New York, New York, ^71^Department of Pathology, Duke University, Durham, North Carolina, ^72^Department of Genome Sciences, University of Washington, Seattle, Washington, ^73^Department of Medicine (Medical Genetics), University of Washington, Seattle, Washington, ^74^Sanders‐Brown Center on Aging, Department Neurology, University of Kentucky, Lexington, Kentucky, ^75^Department of Pathology and Laboratory Medicine, University of California Davis, Sacramento, California, ^76^Department of Biostatistics, Boston University, Boston, Massachusetts, ^77^Department of Ophthalmology, Boston University, Boston, Massachusetts, ^78^University of Pittsburgh Alzheimer's Disease Research Center, Pittsburgh, Pennsylvania, ^79^Department of Neurology, Oregon Health & Science University, Portland, Oregon, ^80^Department of Neurology, Portland Veterans Affairs Medical Center, Portland, Oregon, ^81^Department of Pathology and Laboratory Medicine, University of California Irvine, Irvine, California, ^82^Department of Neurology, Boston University, Boston, Massachusetts, ^83^Department of Pathology, Boston University, Boston, Massachusetts, ^84^Department of Neuropsychology, University of California San Francisco, San Francisco, California, ^85^Department of Molecular & Medical Genetics, Oregon Health & Science University, Portland, Oregon, ^86^Department of Epidemiology, University of Washington, Seattle, Washington, ^87^Department of Neurobiology and Behavior, University of California Irvine, Irvine, California, ^88^Group Health Research Institute, Group Health, Seattle, Washington, ^89^Department of Pathology, University of Washington, Seattle, Washington, ^90^Department of Psychiatry and Behavioral Sciences, University of Washington School of Medicine, Seattle, Washington, ^91^Department of Pathology, University of Michigan, Ann Arbor, Michigan, ^92^Department of Psychiatry, Johns Hopkins University, Baltimore, Maryland, ^93^Department of Preventive Medicine, University of Southern California, Los Angeles, California, ^94^Department of Medicine ‐ Pulmonary, New York University, New York, New York, ^95^Department of Neurology, University of Miami, Miami, Florida, ^96^Department of Pathology, University of California San Diego, La Jolla, California, ^97^School of Nursing Northwest Research Group on Aging, University of Washington, Seattle, Washington, ^98^Department of Neurology, Northwestern University Feinberg School of Medicine, Chicago, Illinois, ^99^Department of Pathology, University of Southern California, Los Angeles, California, ^100^Department of Neurology, Washington University, St. Louis, Missouri, ^101^Arizona Alzheimer's Consortium, Phoenix, Arizona, ^102^Department of Psychiatry, University of Arizona, Phoenix, Arizona, ^103^Banner Alzheimer's Institute, Phoenix, Arizona, ^104^Alzheimer's Disease Center, New York University, New York, New York, ^105^Gertrude H. Sergievsky Center, Columbia University, New York, New York, ^106^Department of Neurology, Columbia University, New York, New York, ^107^Tanz Centre for Research in Neurodegenerative Disease, University of Toronto, Toronto, Ontario, Canada, ^108^Department of Neurology, University of Texas Southwestern, Dallas, Texas, ^109^Department of Radiology and Imaging Sciences, Indiana University, Indianapolis, Indiana, ^110^Department of Pathology (Neuropathology), Rush University Medical Center, Chicago, Illinois, ^111^Department of Psychiatry, University of Southern California, Los Angeles, California, ^112^Cambridge Institute for Medical Research and Department of Clinical Neurosciences, University of Cambridge, Cambridge, ^113^Center for Human Genetics and Research, Department of Molecular Physiology and Biophysics, Vanderbilt University, Nashville, Tennessee, ^114^Department of Pathology, Johns Hopkins University, Baltimore, Maryland, ^115^Sanders‐Brown Center on Aging, Department of Anatomy and Neurobiology, University of Kentucky, Lexington, Kentucky, ^116^Department of Pathology & Laboratory Medicine, University of California Los Angeles, Los Angeles, California, ^117^Taub Institute on Alzheimer's Disease and the Aging Brain, Department of Pathology, Columbia University, New York, New York, ^118^Department of Psychiatry, Northwestern University Feinberg School of Medicine, Chicago, Illinois, ^119^Department of Psychiatry & Behavioral Sciences, Duke University, Durham, North Carolina, ^120^Department of Pathology, Oregon Health & Science University, Portland, Oregon, ^121^Evelyn F. McKnight Brain Institute, Department of Neurology, Miller School of Medicine, University of Miami, Miami, Florida.

## ADGC Co‐Authors

Marilyn S. Albert^1^, Roger L. Albin^2‐4^, Liana G. Apostolova^5^, Steven E. Arnold^6^, Sanjay Asthana^7‐9^, Craig S. Atwood^7,9^, Clinton T. Baldwin^10^, Robert Barber^11^, Michael M. Barmada^12^, Lisa L. Barnes^13,14^, Thomas G. Beach^15^, James T. Becker^16^, Gary W. Beecham^17,18^, Duane Beekly^19^, David A. Bennett^13,20^, Eileen H. Bigio^21,22^, Thomas D. Bird^23,24^, Deborah Blacker^25,26^, Bradley F. Boeve^27^, James D. Bowen^28^, Adam Boxer^29^, James R. Burke^30^, Joseph D. Buxbaum^31‐33^, Nigel J. Cairns^34^, Laura B. Cantwell^35^, Chuanhai Cao^36^, Chris S. Carlson^37^, Cynthia M. Carlsson^8^, Regina M. Carney^38^, Steven L. Carroll^39^, Helena C. Chui^40^, David G. Clark^41^, Jason Corneveaux^42^, David H. Cribbs^44^, Elizabeth A. Crocco^38^, Carlos Cruchaga^45^, Philip L. De Jager^46,47^, Charles DeCarli^48^, Steven T. DeKosky^49^, F. Yesim Demirci^12^, Malcolm Dick^50^, Ranjan Duara^51^, Denis Evans^52^, Kelley M. Faber^53^, Kenneth B. Fallon^39^, Martin R. Farlow^54^, Steven Ferris^55^, Tatiana M. Foroud^53^, Matthew P. Frosch^56^, Douglas R. Galasko^57^, Mary Ganguli^58^, Marla Gearing^59,60^, Daniel H. Geschwind^61^, Bernardino Ghetti^62^, John R. Gilbert^17,18^, Jonathan D. Glass^63^, Alison M. Goate^45^, Robert C. Green^64^, John H. Growdon^65^, Hakon Hakonarson^66^, Ronald L. Hamilton^67^, Kara L. Hamilton‐Nelson^17^, John Hardy^68^, Lindy E. Harrell^41^, Elizabeth Head^69^, Lawrence S. Honig^70^, Matthew J. Huentelman^42^, Christine M. Hulette^71^, Bradley T. Hyman^65^, Gail P. Jarvik^72,73^, Gregory A. Jicha^74^, Lee‐Way Jin^75^, Gyungah Jun^10,76,77^, M. Ilyas Kamboh^12,78^, Anna Karydas^29^, Jeffrey A. Kaye^79,80^, Ronald Kim^81^, Edward H. Koo^57^, Neil W. Kowall^82,83^, Joel H. Kramer^84^, Patricia Kramer^79,85^, Walter A. Kukull^86^, Brian W. Kunkle^17^, Frank M. LaFerla^87^, James J. Lah^63^, Eric B. Larson^43,88^, James B. Leverenz^89^, Allan I. Levey^63^, Ge Li^90^, Andrew P. Lieberman^91^, Chiao‐Feng Lin^35^, Oscar L. Lopez^78^, Kathryn L. Lunetta^76^, Constantine G. Lyketsos^92^, Wendy J. Mack^93^, Daniel C. Marson^41^, Eden R. Martin^17,18^, Frank Martiniuk^94^, Deborah C. Mash^95^, Eliezer Masliah^57,96^, Wayne C. McCormick^43^, Susan M. McCurry^97^, Andrew N. McDavid^37^, Ann C. McKee^82,83^, Marsel Mesulam^22,98^, Bruce L. Miller^29^, Carol A. Miller^99^, Joshua W. Miller^75^, Thomas J. Montine^89^, John C. Morris^34,100^, Jill R. Murrell^53,62^, Amanda J. Myers^38^, Adam C. Naj^35^, John M. Olichney^48^, Amanda Partch^35^, Henry L. Paulson^2^, William Perry^17^, Elaine Peskind^90^, Aimee Pierce^44^, Wayne W. Poon^50^, Huntington Potter^36^, Joseph F. Quinn^79^, Ashok Raj^36^, Murray Raskind^90^, Eric M. Reiman^42,101‐103^, Barry Reisberg^55,104^, Christiane Reitz^70,105,106^, John M. Ringman^5^, Erik D. Roberson^41^, Ekaterina Rogaeva^107^, Howard J. Rosen^29^, Roger N. Rosenberg^108^, Mark A. Sager^8^, Mary Sano^32^, Andrew J. Saykin^53,109^, Julie A. Schneider^13,110^, Lon S. Schneider^40,111^, William W. Seeley^29^, Amanda G. Smith^36^, Joshua A. Sonnen^89^, Salvatore Spina^62^, Peter St George‐Hyslop^107,112^, Robert A. Stern^82^, Rudolph E. Tanzi^65^, Tricia A. Thornton‐Wells^113^, John Q. Trojanowski^35^, Juan C. Troncoso^114^, Debby W. Tsuang^24,90^, Otto Valladares^35^, Vivianna M. Van Deerlin^35^, Linda J. Van Eldik^115^, Badri N. Vardarajan^70,105,106^, Harry V. Vinters^5,116^, Jean Paul Vonsattel^117^, Li‐San Wang^35^, Sandra Weintraub^22,118^, Kathleen A. Welsh‐Bohmer^30,119^, Jennifer Williamson^70^, Sarah Wishnek^17^, Randall L. Woltjer^120^, Clinton B. Wright^121^, Chang‐En Yu^43^, Lei Yu^13^.

## ADGC Declarations of Interest

T.D.B. received licensing fees from and is on the speaker's bureau of Athena Diagnostics, Inc. M.R.F. receives research funding from BristolMyersSquibb Company, Danone Research, Elan Pharmaceuticals, Inc., Eli Lilly and Company, Novartis Pharmaceuticals Corporation, OctaPharma AG, Pfizer Inc., and Sonexa Therapeutics, Inc.; Receives honoraria as scientific consultant from Accera, Inc., Astellas Pharma US Inc., Baxter, Bayer Pharmaceuticals Corporation, BristolMyersSquibb, Eisai Medical Research, Inc., GE Healthcare, Medavante, Medivation, Inc., Merck & Co., Inc., Novartis Pharmaceuticals Corp., Pfizer, Inc., Prana Biotechnology Ltd., QR Pharma., Inc., The sanofi‐aventis Group, and Toyama Chemical Co., Ltd.; and is speaker for Eisai Medical Research, Inc., Forest Laboratories, Pfizer Inc. and Novartis Pharmaceuticals Corporation. A.M.G. has research funding from AstraZeneca, Pfizer and Genentech, and has received remuneration for giving talks at Pfizer and Genentech. R.C.P. is on the Safety Monitory Committee of Pfizer, Inc. (Wyeth) and a consultant to the Safety Monitoring Committee at Janssen Alzheimer's Immunotherapy Program (Elan), to Elan Pharmaceuticals, and to GE Healthcare. R.E.T. is a consultant to Eisai, Japan in the area of Alzheimer's genetics and a shareholder in, and consultant to Pathway Genomics, Inc, San Diego, CA.

## Supporting information


**Figure S1. QQ plots**. Joint analysis results’ QQ plots and genomic inflation factors are shown for (A) unstratified, (B) H2 non‐carrier, and (C) H2 carrier analyses. All analyses included cohorts, age, sex, PC1‐3, and *APOE* as covariates. QQ plots display observed versus expected *P* ‐values given the number of statistical tests performed for each SNP. The number of SNPs shown was LD pruned for visualization. A red diagonal represents the expected distribution. Points to the left of the diagonal represent associations that are more significant than expected. Genomic inflation (λ) estimates were obtained for each data set.

**Figure S2. Manhattan plot of unstratified joint association analysis**. *P* values from the analytic model without *APOE* as a covariate is shown. The threshold for genome‐wide significance (*P* < 5E10‐8) is indicated by the red line and the threshold for trending significance (*p* < 1E‐5) is indicated by the blue line. Loci with *p* < 1E‐5 are annotated as mustard yellow.

**Figure S3. Forest plot of discordant loci**. Results are shown for the discordant loci depicted in Table 2 for joint association analysis excluding *APOE*. (A) Discordant loci with *P* ≤ 1E‐5 in H2 non‐carriers. (B) Discordant loci with *P* ≤ 1E‐5 in H2 carriers. Point size of odds ratio is weighted by the N of each group. Chromosome, nearest gene name(s), and most significant variant at each of the discordant loci are shown at the top of the figures. ADGC cohort names are shown on the left; and their corresponding odds ratios (ORs) and 95% confidence intervals (95% CIs) are shown to the right of the figures. Meta‐analysis OR and 95% CI results of the discordant variants are shown on the bottom of each figure.
