# Supplementary figures and images for: MAPT haplotype–stratified GWAS reveals differential association for AD risk variants
Source: Alzheimers Dement. 2020 May 13;16(7):983–1002. doi: 10.1002/alz.12099 (PMC7983911; doi:10.1002/alz.12099)

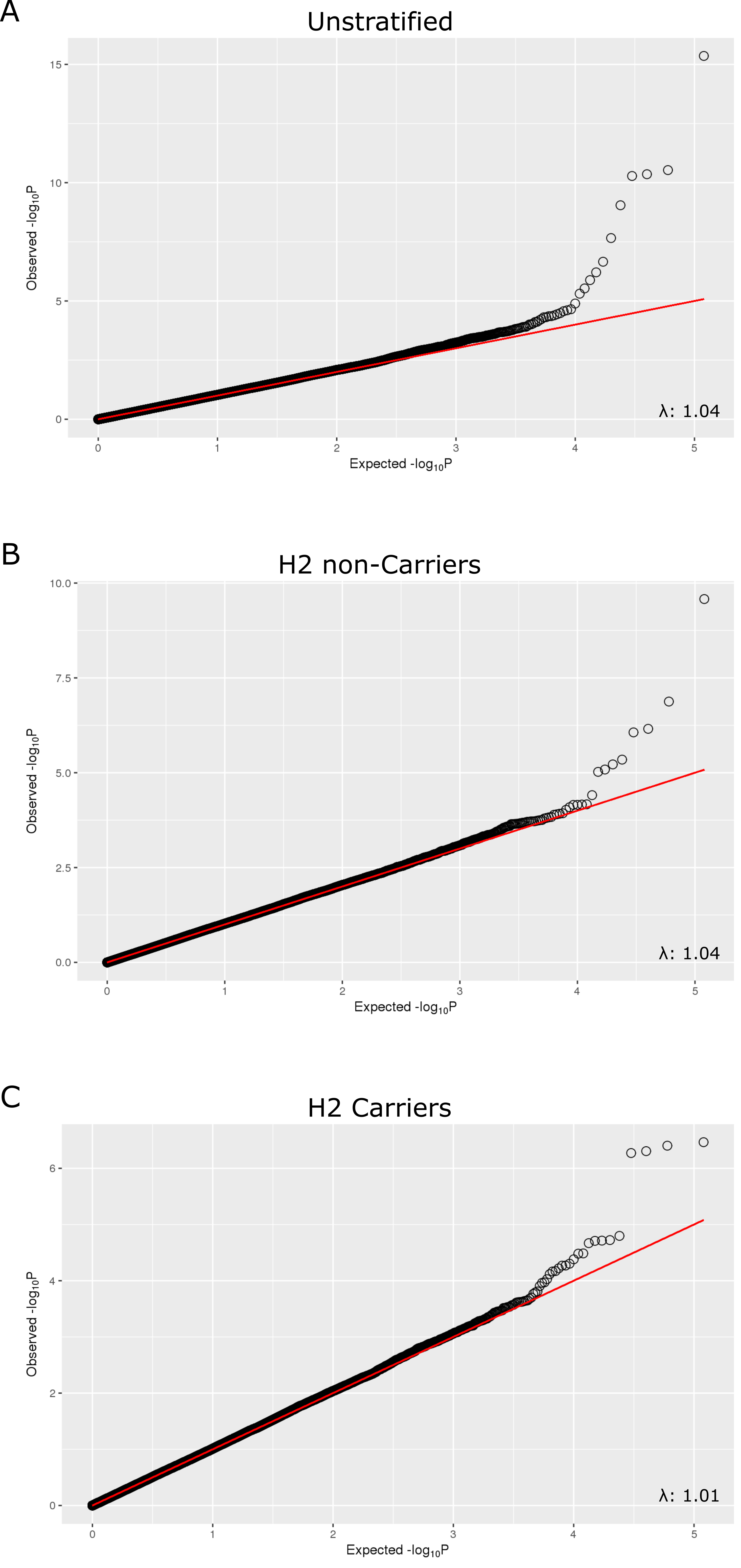

Supplement: Supplementary file 1 — Figure S1. QQ plots. Joint analysis results’ QQ plots and genomic inflation factors are shown for (A) unstratified, (B) H2 non‐carrier, and (C) H2 carrier analyses. All analyses included cohorts, age, sex, PC1‐3, and APOE as covariates. QQ plots display observed versus expected P ‐values given the number of statistical tests performed for each SNP. The number of SNPs shown was LD pruned for visualization. A red diagonal represents the expected distribution. Points to the left of the diagonal represent associations that are more significant than expected. Genomic inflation (λ) estimates were obtained for each data set. [file ALZ-16-983-s001.tiff]

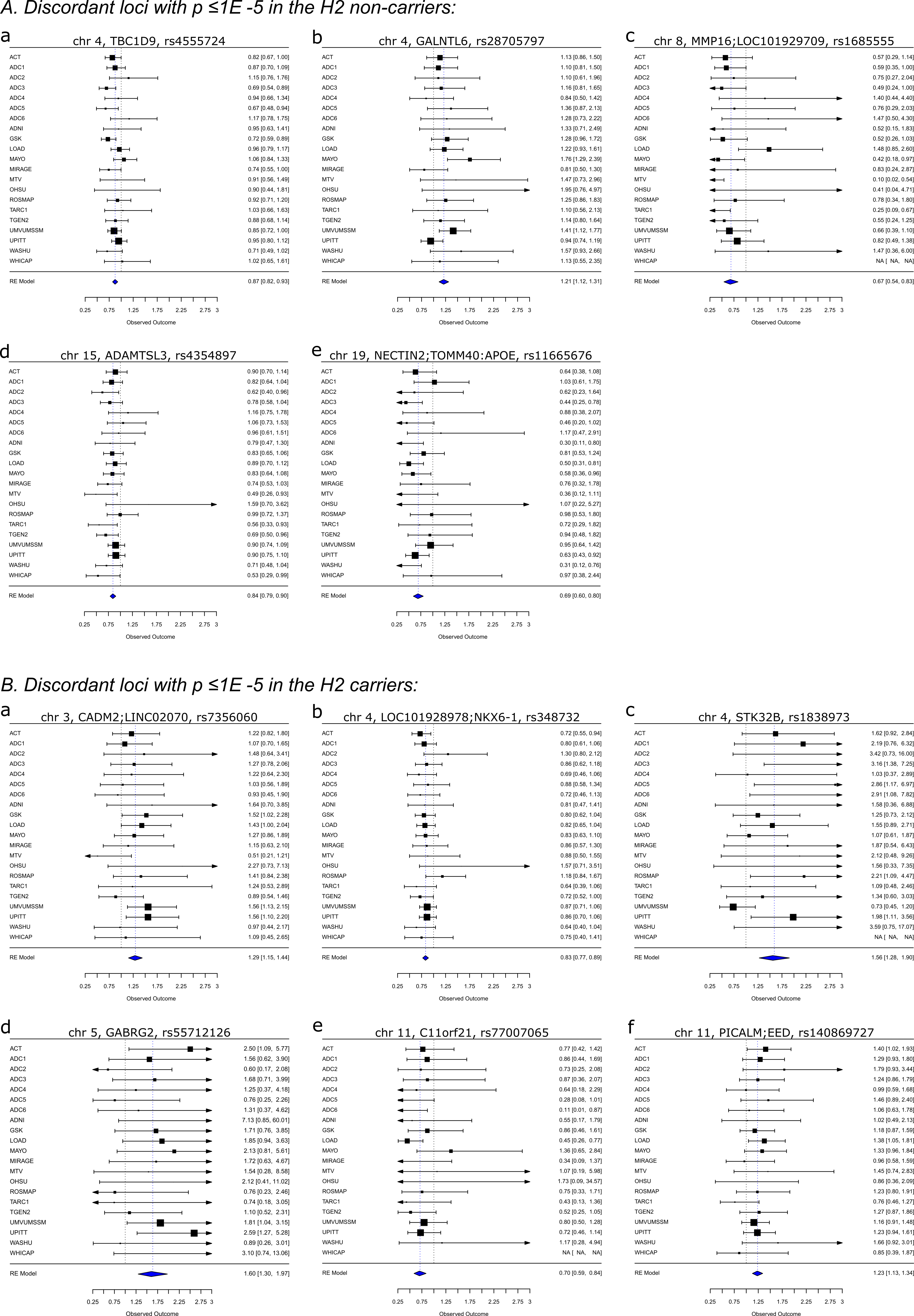

Supplement: Supplementary file 3 — Figure S3. Forest plot of discordant loci. Results are shown for the discordant loci depicted in Table 2 for joint association analysis excluding APOE. (A) Discordant loci with P ≤ 1E‐5 in H2 non‐carriers. (B) Discordant loci with P ≤ 1E‐5 in H2 carriers. Point size of odds ratio is weighted by the N of each group. Chromosome, nearest gene name(s), and most significant variant at each of the discordant loci are shown at the top of the figures. ADGC cohort names are shown on the left; and their corresponding odds ratios (ORs) and 95% confidence intervals (95% CIs) are shown to the right of the figures. Meta‐analysis OR and 95% CI results of the discordant variants are shown on the bottom of each figure. [file ALZ-16-983-s003.tiff]
